# A review of the impact and outcomes of the 2004 U.S. preventive services task force recommendation against scoliosis screening

**DOI:** 10.1186/1748-7161-8-S2-O11

**Published:** 2013-09-18

**Authors:** Joseph O'Brien, Joanna Chowanska

**Affiliations:** 1 SMARTT Institute - National Scoliosis Foundation, Stoughton, MA, USA

## Background

In 2004, the U.S. Preventive Services Task Force (USPSTF) recommended against the routine screening of asymptomatic adolescents for idiopathic scoliosis (IS). The stated rationale in the Summary of the Recommendation was “the USPSTF concluded that the harms of screening adolescents for idiopathic scoliosis exceed the potential benefits.” [1] Since that time, at least five states have removed scoliosis screening from their school health requirements.

## Purpose

The purpose of this study is to present the preliminary results of a review of the evidence based outcomes related to the USPSTF’s recommendation against scoliosis screening in the U.S.

## Methods

We analyzed the Healthcare Cost and Utilization Project (HCUP) database[2] for hospital discharges and costs for ICD 9–737.30 “Idiopathic Scoliosis” for the period 2003–2010, in total and by age group and gender. Our review included a comparative analysis of both the nation as a whole as well as states with legislated screening (SS) and states without legislated screening (NS).

## Results

Nationwide hospital discharges for IS were 6,579 in 2003 compared to 13,531 in 2010, an increase of 105.7%. Mean charges for treatment for IS in 2003 were $88,875 compared to $166,344 in 2010, an increase of 87.2%. According to the state data for this same period, total hospital discharges for SS increased 60.4% versus 73.3% for NS (the 18-44 year-old group discharges for SS increased only 5.6% versus 21.1% for NS), and the increase in total mean charges for treatment was 86.6% for SS versus 93.4% for NS.

## Conclusions and discussion

Since publication of the USPSTF’s recommendation against scoliosis screening, the U.S. has seen an increasing nationwide trend in both the number of hospital discharges of IS patients and the mean charges for their treatment. In addition, the increase in the number of discharges was higher in NS states compared to SS states, and the increase in total charges for treatment was higher in NS states compared to SS states. The USPSTF recommendation has reduced the number of screening programs in the U.S., consistent with the intent of their policy. However, the HCUP data suggest that contrary to the USPSTF’s stated rationale, the reduction in scoliosis screening appears to have caused more harm, not less, for IS patients and society as a whole, in terms of an increase in the volume and economic burden of scoliosis surgeries. Further studies with additional databases and statistical analyses are needed to address the limitations of the HCUP database and this preliminary review.
